# Physiological plasticity in a successful invader: rapid acclimation to cold occurs only in cool-climate populations of cane toads (*Rhinella marina*)

**DOI:** 10.1093/conphys/cox072

**Published:** 2018-01-23

**Authors:** Samantha M McCann, Georgia K Kosmala, Matthew J Greenlees, Richard Shine

**Affiliations:** School of Life and Environmental Sciences, University of Sydney, Room 442, Heydon-Laurence Building (A08) Science Road, New South Wales 2006, Australia

**Keywords:** Acclimation, alien species, *Bufo marinus*, rapid evolution, thermal biology

## Abstract

Physiological plasticity may facilitate invasion of novel habitats; but is such plasticity present in all populations of the invader or is it elicited only by specific climatic challenges? In cold-climate areas of Australia, invasive cane toads (*Rhinella marina*) can rapidly acclimate to cool conditions. To investigate whether this physiological plasticity is found in all invasive cane toads or is only seen in cool climates, we measured the acclimation ability of toads from across Australia and the island of Hawai’i. We collected toads from the field and placed them at either 12 or 24°C for 12 h before measuring their righting response as a proxy for critical thermal minimum (CTmin). Toads from the coolest Australian region (New South Wales) demonstrated plasticity (as previously reported), with exposure to 12°C (vs. 24°C) decreasing CTmin by 2°C. In toads from other Australian populations, CTmins were unaffected by our thermal treatments. Hawai’ian toads from a cool, wet site also rapidly acclimated to cool conditions, whereas those from warmer and drier Hawai’ian sites did not. Thermal plasticity has diverged among populations of invasive cane toads, with rapid acclimation manifested only in two cool-climate populations from widely separated sites. Predictions about the potential range of invasive species thus must consider the possibility of geographic (intraspecific) heterogeneity in thermal plasticity; data from other parts of the species’ range may fail to predict levels of plasticity elicited by thermal challenges.

## Teaser

Invasive cane toads have been present in Australia and Hawai’i for ~80 years. Toads have invaded sites in each location that are cooler than their native range. These populations have both developed the ability to rapidly acclimate to cold conditions, decreasing their critical minimum temperature over just 12 h.

## Introduction

Understanding how invasive species flourish in particular environments is essential for accurately predicting the invader’s impact and informing management. Mathematical models often use information on ‘average’ characteristics of a species (e.g. clutch size, upper and lower thermal limits for activity, habitat preferences) in combination with fine-scale climatic data, to predict the potential extent of a biological invasion (Kearney and Porter, 2009; Elith *et al.*, 2010). However, that approach neglects the possibility of phenotypic divergence among spatially separated populations. Rapid shifts in phenotypic traits may occur when an invader encounters novel environments (Phillips *et al.*, 2007; Whitney and Gabler, 2008; Kolbe *et al.*, 2010; Tingley *et al.*, 2012) either via plasticity (direct modifications in response to environmental factors: Agrawal, 2001) or through the evolution of trait values over time (Prentis *et al.*, 2008) or through space (spatial sorting: Shine *et al.*, 2011). Plasticity can involve many traits (morphology, behaviour, physiology), and take many forms, including developmental plasticity (non-reversible) and reversible acclimation (Piersma and Drent, 2003). Changes can also occur over many timescales, for example rapid ‘cold-hardening’ in plants in response to frost (Beck *et al.*, 2004) or more gradual physiological adjustment (e.g. seasonal thermal acclimatization: Piersma and van Gils, 2010). Incorporating information on variation and flexibility of traits may improve the accuracy of predictions of invader spread.

Invasion success is affected by many environmental and historical factors (e.g. similarity of the climate to that in the species’ native range, and frequency of introduction events: Kolar and Lodge, 2001; Jeschke and Strayer, 2006; Hayes and Barry, 2008; Mahoney *et al.*, 2015), but also by species-level traits. Many attempts have been made to characterize phenotypic correlates of successful invasion, in order to predict the invasive ability of a particular species, or their potential to invade a particular environment (Kolar and Lodge, 2001; Jeschke and Strayer, 2006; Hayes and Barry, 2008; Klonner *et al.*, 2016). One of the most significant such traits may be plasticity, that is, the ability to adjust phenotypic characteristics in response to local environments (Sol and Lefebvre, 2000; Geng *et al.*, 2007; Wright *et al.*, 2010; Li *et al.*, 2016).

The cane toad (*Rhinella marina*) is one of the world’s most successful invasive species (Lowe *et al.*, 2000). Although native to Central and South America, this large bufonid anuran has been introduced to >40 countries across the globe, and now inhabits a wide range of environments (Lever, 2001). Cane toads were introduced to northeastern Australia (from French Guiana, via Hawai’i) in 1935. Northeastern Australia is humid and tropical, and provides climatic conditions relatively similar to those within the species’ native range (Tingley *et al.*, 2012). Cane toads spread rapidly westwards, reaching Darwin in the Northern Territory in 2005, and entering Western Australia in 2010. The southern front of the invasion reached the New South Wales border in 1978, and has since moved slowly southward, while several translocations have further extended the southern distribution (NSW: Seabrook, 1991; Estoup *et al.*, 2004). Montane sites in this latter region are colder than over most of the species’ native range in tropical America, posing novel thermal challenges (Newell, 2011; McCann *et al.*, 2014; Tingley *et al.*, 2014). One mechanism contributing to the invader’s unexpected success in this cool area is the toads’ ability to rapidly adjust their lower thermal tolerances in response to a few hours’ exposure to cool conditions (McCann *et al.*, 2014; Winwood-Smith *et al.*, 2015). However, we do not know whether this ability to acclimate is a trait of all cane toads, or is only seen in populations that experience cool conditions. To answer this question, we quantified the acclimation ability of cane toads from across Australia, and also from populations in Hawai’i. Cane toads have been present in Hawai’i and Australia for similar periods (~80 years) and Hawai’i was the source of the toads brought to Australia. Both Hawai’i and Australia impose a wide range of thermal conditions, providing a robust opportunity to look for associations between climate and thermal plasticity among invasive populations.

## Materials and methods

### Australian field sites

**Figure 1:**
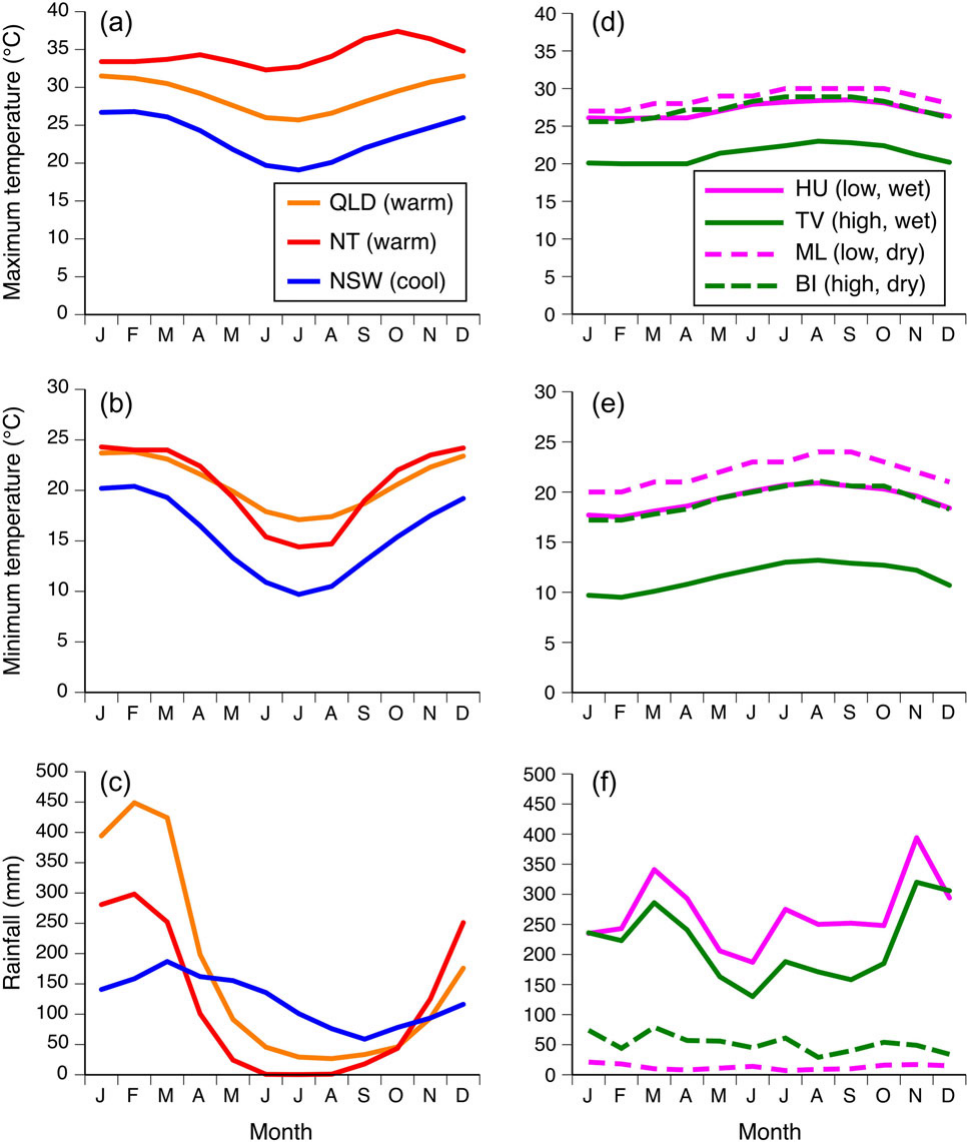
Monthly average maximum temperature (°C), minimum temperature (°C) and rainfall (mm) at our sites in Australia (**a**–**c**) and Hawai’i (**d**–**f**) over ~20 years. Australia; blue line = New South Wales (NSW), red line = Northern Territory (NT), orange line = Queensland (QLD). Hawai’i; pink solid line = University of Hawaii (HU), pink dashed line = Mauna Lani (ML), green solid line = Tom’s Farm near volcano (TV), green dashed line = Big Island Country Club (BI). See Materials and methods for sources

We tested the lower thermal tolerance of adult cane toads from Cairns, Queensland (QLD: three sites, May 2016), Darwin, Northern Territory (NT: three sites, November 2015), and Yamba, northeastern New South Wales (NSW: two sites, February 2016; see Table 1 for site details). All Australian sites were <30 m in elevation (above sea level, asl), with mean annual precipitation ranging from 1391 to 2003 mm, which is highly seasonal in the more arid sites (Fig. 1).

**Table 1:** Sites in Australia and Hawai’i in which cane toads (*Rhinella marina*) were collected and tested. The Table shows number of toads, sexes, mean mass (g, ±SE), snout-urostyle length (SUL; mm ±SE), GPS location, elevation (m, asl) and dates that toads were tested

Country, state or county	Site	Total # toads	Male	Female	Mass (g ± SE)	SUL (mm ± SE)	GPS coordinates	Elevation (m, asl)	Dates tested
**Australia**									
New South Wales (NSW)	Woombah	22	13	9	49.0 ± 2.8	82.9 ± 1.5	−29.354637, 153.253596	26	February 2016
	Yamba	24	13	11	90.6 ± 4.8	93.8 ± 1.5	−29.440139, 153.361000	29	February 2016
Northern Territory (NT)	Leaning Tree Lagoon (LTL)	30	28	2	141.4 ± 5.2	115.6 ± 1.2	−12.711970, 131.419141	1	November 2015
	Middle Point (MP)	28	10	18	205.9 ± 13.0	123.2 ± 2.1	−12.578218, 131.315515	1	November 2015
	Mary River Park (MRP)	29	12	17	158.0 ± 6.8	118.3 ± 1.4	−12.904739, 131.650443	1	November 2015
Queensland (QLD)	Smithfield	24	15	9	104.5 ± 5.2	99.9 ± 1.6	−16.825886, 145.688713	19	May 2016
	Yorkey’s Knob	26	7	19	138.1 ± 13.4	108.0 ± 3.1	−16.829291, 145.707909	10	May 2016
	Cairns Botanic Gardens	28	16	12	112.5 ± 5.9	103.2 ± 1.4	−16.899718, 145.747294	10	May 2016
**Hawai’i**									
Big Island	Hilo University (HU)	20	8	12	103.3 ± 8.4	100.6 ± 2.3	19.696853, −155.081501	40	June 2015
	Tom’s Farm near Volcano (TV)	22	19	3	68.9 ± 3.8	91.6 ± 1.5	19.549037, −155.136774	571	June 2015
	Mauna Lani (ML)	20	12	8	148.8 ± 10.6	116.8 ± 2.1	19.941369, −155.859498	10	June 2015
	Big Island Country Club (BI)	20	15	5	99.9 ± 5.1	103.5 ± 1.5	19.819891, −155.836336	630	June 2015

### Hawai’ian field sites

We tested the lower thermal tolerance of adult cane toads from four sites on the Big Island of Hawai’i in June 2015. In the Hawai’ian Islands, even nearby areas may differ strongly in wind speed, cloudiness and rainfall (Sanderson, 1993). The porous volcanic soil exacerbates effects of rainfall variation on toad ecology (Ward-Fear *et al.*, 2016). Our sites comprised a low-elevation (40 m, asl) area on the wet (windward) side of the island (University of Hawai’i at Hilo, HU), a high-elevation (570 m asl) area also on the wet side (Tom’s Farm near Volcano, TV), a high-elevation (630 m asl) area on the dry (leeward) side of the island (Big Island Country Club, BI), and a low-elevation (10 m asl) area on the dry side (Mauna Lani Resort, ML) (Table 1). Average annual precipitation ranges from 2607 to 3218 mm at our ‘wet’ sites (TV and HU, respectively), and 156 to 622 mm at our ‘dry’ sites (ML and BI; Fig. 1).

### Measurements of critical thermal minimum

We collected 20–30 adult toads (Table 1) of mixed sexes from each field site at night, and immediately transported them back to a nearby field laboratory. Within 1 h of collection we placed each toad into a separate calico bag, which was then placed inside a 1 L plastic container. Half of the toads were then put into a refrigerator set to 12°C, and half into a 24°C room. Toads were left for 12 h (overnight), with temperatures checked regularly throughout this time. After 12 h we measured the lower thermal tolerance of each toad by using loss of righting response as a proxy for critical thermal minimum (CTmin). To do this, we measured the initial body temperature of a toad by inserting a temperature probe 1 cm into its cloaca. We then placed the toad inside a closed 1 L plastic container, which was placed into an insulated box filled with ice. As the toad cooled we removed it from the box every 10 min, recorded its body temperature, and placed it on its back on a flat substrate. If the toad righted itself (turned over) within 30 s, it was returned to the icebox for a further 10 min then retested. This was repeated until the toad no longer righted itself within 30 s. The temperature of the toad at this time was recorded as its CTmin. This measurement (a commonly used index of CTmin) represents a biologically valid and ethically acceptable measure of an animal’s ability to function (Cowles and Bogert, 1944; Spellerberg, 1972; Kolbe *et al.*, 2010). Toads from both thermal treatments were tested simultaneously to eliminate any confounding effects associated with time of testing.

### Analysis of data

We tested for an effect of acclimation temperature on the CTmin of toads in Australia by running a two-way ANOVA in SPSS (IBM, Armonk, New York) with factors ‘acclimation temperature’ and ‘location’, and ‘site’ nested within location as a random factor. We included toad body mass and cooling rate as covariates as these can influence CTmin measurements (Kolbe *et al.*, 2010). The data for Hawai’ian toads showed a significant difference in cooling rates between sites, preventing us from comparing absolute CTmin values between sites. Including ‘cooling rate’ as a covariate in this case would assume both linearity of effect, and consistency of effect among sites. To overcome that problem we analysed data from each Hawai’ian site separately, focusing on the effects of acclimation treatments within toads from the same site (cooling rates were consistent between treatments within sites). To do this we ran an ANOVA with factor ‘acclimation treatment’ and variable ‘CTmin’ for each site, and included ‘toad body mass’ and ‘cooling rate’ as covariates. Measurements from ML were square-root transformed before analysis due to heterogeneity of variances (Levene’s test: *F*_1,19_ = 4.71, *P* < 0.05).

### Climate summaries

Monthly average maximum temperature (°C), minimum temperature (°C) and rainfall (mm) were summarized from approximately the past 20 years for each site by combining data from a range of sources. Australian data were sourced from the Bureau of Meteorology (www.bom.gov.au). Hawai’ian data were combined from ‘US climate data’ (www.usclimatedata.com), ‘My Weather’ (www.myweather2.com) and ‘Weather’ (www.weather.com).

## Results

### Cane toads from Australia

**Figure 2:**
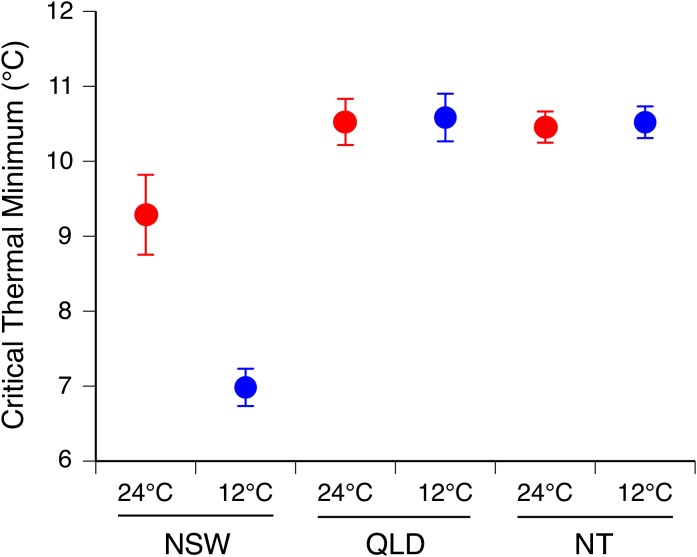
Critical thermal minima of cane toads (*Rhinella marina*), tested after 12 h of acclimation to either cold (12°C, blue circles) or hot (24°C, red circles) conditions. Toads were collected at multiple sites from three locations in Australia (NSW: Yamba and Woombah, *n* = 24 and 22, respectively, QLD: Smithfield, Yorkey’s Knob and Cairns Botanic Gardens, *n* = 24, 26 and 28, respectively, and NT: Middle Point, Leaning Tree Lagoon and Mary River Park, *n* = 28, 30 and 29, respectively). Data for sites within each location have been combined in this figure. Critical thermal minimum was quantified as the temperature at which the toads lost their ability to right themselves after being turned over. The graph shows mean values ± SE

After exposure to either 12 or 24°C for 12 h, a toad’s CTmin was affected by a significant interaction between acclimation temperature and geographic origin of toads (QLD, NT or NSW; *F*_2,199_ = 7.87, *P* = 0.001), with no significant difference among sites within locations (*F*_5,199_ = 0.65, *P* = 0.66). CTmins of toads from QLD and NT were unaffected by acclimation treatment, but NSW toads that were acclimated to 12°C exhibited a CTmin 2°C lower than did NSW toads acclimated to 24°C (Fig. 2).

### Cane toads from Hawai’i

**Figure 3:**
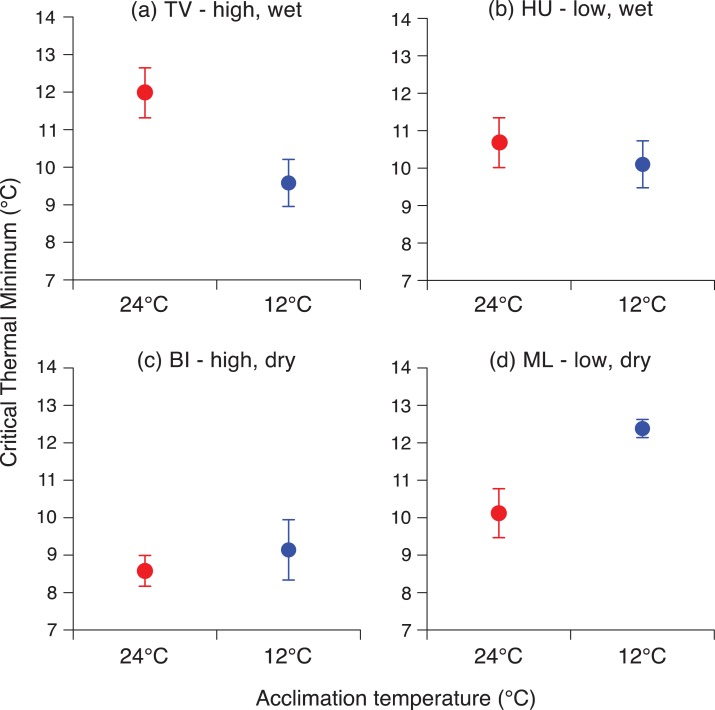
Critical thermal minima of cane toads (*Rhinella marina*), tested after 12 h of acclimation to either cold (12°C, blue circles) or hot (24°C, red circles) conditions. Toads were collected from four sites within the Big Island of Hawai’i: (**a**) TV = Tom’s Farm near volcano, (**b**) HU = Hilo University, (**c**) BI = Big Island Country Club and (**d**) ML = Mauna Lani, *n* = 22, 20, 20 and 20, respectively. Critical thermal minimum was quantified as the temperature at which the toads lost their ability to right themselves after being turned over. The graphs show mean values ± SE

In Hawai’i, acclimation responses varied among sites. Toads from TV (high, wet) showed a similar response to those from NSW, with individuals acclimated to 12°C exhibiting a CTmin 2°C lower than those acclimated to 24°C (*F*_1,21_ = 7.04, *P* = 0.01; Fig. 3a). Toads from HU (low, wet) and BI (high, dry) showed no such difference in CTmins between 12 and 24°C treatments (HU: *F*_1,19_ = 1.36, *P* = 0.26; BI: *F*_1,19_ = 0.01, *P* = 0.91; Fig. 3b and c). At ML (low, dry) however, toads showed the opposite pattern to TV, with toads acclimated to 12°C displaying a CTmin 2°C higher than those acclimated to 24°C (*F*_1,19_ = 6.44, *P* = 0.02; Fig. 3d).

## Discussion

We documented rapid acclimation to cool conditions in two populations of invasive cane toads living in cool, wet climates. No such acclimation was evident in populations from warmer or drier areas. Although thermal acclimation is widespread (Piersma and van Gils, 2010), we are not aware of any previous reports of such a dramatic shift in this ability within the short timeframe (80 years) that toads have been present in both Australia and Hawai’i. Our design does not allow us to say whether the thermal plasticity of toads in cool climates is genetically coded (i.e. due to local adaptation), or is a manifestation of developmental plasticity (i.e. long-term exposure to cold conditions causes a toad to develop the ability to shift its CTmin accordingly: Beaman *et al.*, 2016). This question could be resolved by running a ‘common garden’ experiment, testing the thermal plasticity of toads from different populations that have been raised under standard conditions.

This ‘common garden’ approach has been applied to investigate other traits of cane toads in Australia, and has shown that the substantial phenotypic differences between toads from different areas are underpinned by heritable factors as well as phenotypic plasticity (Shine, 2012; Rollins *et al.*, 2015). For example, toads at the western invasion front have wider forelimbs, narrower hind limbs and more compact skulls than conspecifics in long-colonized areas of eastern Australia, and these traits are heritable under common garden conditions (Hudson *et al.*, 2016a). Similarly, the offspring of invasion-front toads inherit their parents’ propensity for sustained and unidirectional dispersal (Phillips *et al.*, 2010; Brown *et al.*, 2014), and distinctive patterns of immune response (Llewellyn *et al.*, 2011; Brown *et al.*, 2015). In contrast, differences among toad populations in climbing ability seem to be driven primarily by local conditions rather than genetic factors (Hudson *et al.*, 2016b). Geographic variation in phenotypic traits of cane toads may enable the animals to function well under spatially variable challenges, increasing invasion success. Our result adds another kind of trait to this expanding list—the ability of cane toads to exploit cold environments is facilitated by rapid thermal acclimation.

The relationship between acclimation ability and climate among Australian sites is clear: toads in cool areas exhibit a rapid thermal response, whereas toads in warmer areas lack this ability. In Hawai’i, however, toads from one high-elevation, assumedly ‘cool’ site exhibited rapid acclimation whereas conspecifics from another such site did not. That paradox may be due to local topography, and its influence on climatic conditions. Annual rainfall is high at one site (2607 mm at TV) and low at the other (635 mm at BI). Our climatic data demonstrate that relationship between elevation and air temperatures differs between the two sides of the island. TV is cooler than the other three sites year-round, whereas BI experiences a thermal profile much like that of the two low-elevation sites. Soil temperatures are ~3°C lower on the ‘wet’ (windward) side than the ‘dry’ (leeward) side of the Big Island at equal elevations (Nullet *et al.*, 1995), and as ground-dwelling ectotherms, the temperatures most relevant to toads are those at the ground surface (as opposed to air temperature). That geographic difference is driven by evaporation from wet soil, combined with denser vegetation shading the soil surface (Nullet *et al.*, 1995). Thus, toads at our high dry site (BI) experience relatively warm air and soil despite the high elevation, reducing any advantage of rapid thermal acclimation; and that situation may explain why we did not detect such an ability in toads from this location.

The other anomalous result from the Hawai’ian toads tested was that at one site (ML = low, dry), toads showed the opposite pattern than expected—higher (rather than lower) CTmin after exposure to low temperatures. That counter-intuitive pattern was not seen at any other site, nor has it been reported in studies of other anurans, to our knowledge. We speculate that at this site the warm and very dry conditions (~156 mm rainfall annually) result in soil temperatures higher than at all other sites (Nullet *et al.*, 1995), where toads rarely experience temperatures low enough to be thermally challenging. Thus, when placed at 12°C for an extended amount of time (12 h), they were unable to function at all. However toads from ML placed at 24°C were still able to right themselves even at relatively low temperatures.

Our results demonstrate not only the remarkable ability of cane toads to rapidly adjust to cooler thermal conditions (increasing invasion success), but also the need for predictive models of toad spread to consider variation in traits between toad populations. If data on the relevant traits of an invasive species are sourced only from a limited range of populations, without considering geographic heterogeneity, the true ability of invaders to occupy a broad range of environments, and hence their eventual spread, may be grossly underestimated.
